# Craniosynostosis-Associated Fgfr2^C342Y^ Mutant Bone Marrow Stromal Cells Exhibit Cell Autonomous Abnormalities in Osteoblast Differentiation and Bone Formation

**DOI:** 10.1155/2013/292506

**Published:** 2013-05-09

**Authors:** J. Liu, T.-G. Kwon, H. K. Nam, N. E. Hatch

**Affiliations:** ^1^Department of Orthodontics and Pediatric Dentistry, School of Dentistry, University of Michigan, Ann Arbor, MI 48109-1078, USA; ^2^Department of Oral and Maxillofacial Surgery, School of Dentistry, Kyungpook National University, Jung Gu, Daegu, Republic of Korea

## Abstract

We recently reported that cranial bones of Fgfr2^C342Y/+^ craniosynostotic mice are diminished in density when compared to those of wild type mice, and that cranial bone cells isolated from the mutant mice exhibit inhibited late stage osteoblast differentiation. To provide further support for the idea that craniosynostosis-associated Fgfr mutations lead to cell autonomous defects in osteoblast differentiation and mineralized tissue formation, here we tested bone marrow stromal cells isolated from Fgfr2^C342Y/+^ mice for their ability to differentiate into osteoblasts. Additionally, to determine if the low bone mass phenotype of Crouzon syndrome includes the appendicular skeleton, long bones were assessed by micro CT. Fgfr2^C342Y/+^ cells showed increased osteoblastic gene expression during early osteoblastic differentiation but decreased expression of alkaline phosphatase mRNA and enzyme activity, and decreased mineralization during later stages of differentiation, when cultured under 2D *in vitro* conditions. Cells isolated from Fgfr2^C342Y/+^ mice also formed less bone when allowed to differentiate in a 3D matrix *in vivo*. Cortical bone parameters were diminished in long bones of Fgfr2^C342Y/+^ mice. These results demonstrate that marrow stromal cells of Fgfr2^C342Y/+^ mice have an autonomous defect in osteoblast differentiation and bone mineralization, and that the Fgfr2^C342Y^ mutation influences both the axial and appendicular skeletons.

## 1. Introduction

Craniosynostosis is a debilitating pediatric condition characterized by the premature fusion of cranial bones. This fusion leads to high intracranial pressure and abnormal skull and facial shapes presumably resulting from limited growth at fused craniofacial sutures with compensating overgrowth at nonfused cranial sutures [1–5]. Untreated craniosynostosis can lead to blindness, seizures, and death [6–10]. Current treatment options for craniosynostosis and its associated craniofacial abnormalities are limited to surgery with genetic counseling, orthodontic, medical, and social support [11]. Notably, even with an appropriately early and accurate diagnosis, craniosynostosis can carry high morbidity, with some patients requiring multiple surgeries throughout infancy and childhood for treatment of recurring craniosynostosis and normalization of skull and facial shapes [12]. 

It has been known for over a decade that craniosynostosis occurs in association with mutations in the genes for fibroblast growth factor receptors (Fgfr's). Mutations in Fgfr2 cause Apert, Crouzon, Jackson-Weiss, and Pfeiffer craniosynostosis syndromes, while mutations in Fgfr1 cause Pfeiffer syndrome and mutations in Fgfr3 cause Muenke craniosynostosis syndrome and Crouzon syndrome with acanthosis nigricans [13–17]. Numerous prior studies have shown that these craniosynostosis-associated Fgfr mutations lead to a gain of function in terms of Fgfr signaling [18–22]. Yet, despite our extensive knowledge in the genetics underlying syndromic craniosynostosis, the pathogenesis by which mutations in Fgfr's lead to craniosynostosis has yet to be fully elucidated. Because Fgf/Fgfr signaling is an important form of intercellular communication, it has been proposed that craniosynostosis-associated mutations in Fgf receptors lead to craniosynostosis by causing inappropriate signaling to cranial cells from neighboring tissues such as the dura mater [23–25]. In contrast, it has also been proposed that craniosynostosis-associated mutations in Fgf receptors lead to intrinsic defects in the behavior of cranial bone cells and tissues [26–28]. Importantly, while the biologic pathogenesis of craniosynostosis remains unknown, the only treatment for craniosynostosis will remain that of surgical intervention. 

To advance our understanding of mechanisms that lead to craniosynostosis, we are investigating the Fgfr2^C342Y/+^ mouse model of Crouzon syndrome. Common features of Crouzon syndrome in humans include coronal suture synostosis with rare pansynostosis, hypertelorism, severe ocular proptosis, strabismus, hypoplastic maxilla, and relative mandibular prognathism [11, 29]. Notably, high rates of stylohyoid ligament calcification and vertebral fusions, as well as the occasional fusion of limb joints, have also been reported [30–32]. Mice carrying the classic Crouzon syndrome associated Fgfr2^C342Y^ mutation were initially reported to have characteristics similar to those of Crouzon syndrome patients including a dome-shaped skull, wide set and proptotic eyes, premature fusion of cranial sutures, and a shortened maxilla [32]. Notably, fusions were also evident between the femur and tibia and between cervical vertebral arches in homozygous mutant mice. The homozygous mutant mice also lacked vertebral body ossification. These findings indicate that the Crouzon syndrome phenotype involves both the axial and appendicular skeletons. The findings also suggest that the bony fusions of Crouzon syndrome occur in the context of diminished eutopic bone formation. 

We recently reported that the frontal bones of Crouzon Fgfr2^C342Y/+^ mice are diminished in bone volume and density when compared to those of wild type mice, and that while frontal bone cells isolated from the mutant mice exhibit increased osteoblastic gene during early stages of osteoblast differentiation, the cells are also diminished in their ability to differentiate into mature osteoblasts *in vitro* [5]. To provide further support for the idea that the Crouzon syndrome associated Fgfr2^C342Y^ mutation leads to intrinsic changes in osteoblast differentiation and bone formation, we isolated cells from Crouzon mice and assayed their ability to differentiate into osteoblasts and form mineralized tissue, as compared to cells isolated from wild type mice. Because cell culture in a three-dimensional matrix supports a more physiologically relevant environment than more traditional two-dimensional cell culture methods [33], we assayed cells in traditional *in vitro* monolayer culture and in a three-dimensional collagenous matrix *in vivo*. Additionally, to determine if the appendicular skeleton is altered in Fgfr2^C342Y/+^ mice, we used microcomputed tomography to quantify parameters of tibial bone quality and quantity. Here we report that the Fgfr2^C342Y^ mutation enhances expression of osteoblastic genes during early stages of differentiation while inhibiting expression of alkaline phosphatase enzyme (AP/Tnap/Alpl/Akp2) and mineralization during later differentiation, in two-dimensional *in vitro* cell culture. Here we also show that the Fgfr2^C342Y^ mutation inhibits the ability of these cells to express Tnap enzyme and form mineralized tissue when allowed to differentiate in a three-dimensional matrix *in vivo*. Additionally, here we report for the first time that the long bones of Fgfr2^C342Y/+^ mice have significantly diminished cortical bone quality and quantity, when compared to those of wild type mice. Together, these results demonstrate that the Crouzon syndrome associated Fgfr2^C342Y^ mutation causes cell autonomous abnormalities in osteoblast differentiation that include enhanced early differentiation but inhibited later expression of Tnap enzyme and mineralization by bone marrow stromal cells. Results also show that Crouzon syndrome is associated with significantly diminished appendicular bone volume and density. 

## 2. Experimental Procedures

### 2.1. Animals

Fgfr2^C342Y/+^ and wild type mice were genotyped as previously described [29]. Briefly, DNA from tail digests was amplified by polymerase chain reaction using 5′-gagtaccatgctgactgcatgc-3′ and 5′-ggagaggcatctctgtttcaagacc-3′ primers. The reaction product was resolved by gel electrophoresis, yielding a 200 base pair band for wild type Fgfr2 and a 300 base pair band for Fgfr2^C342Y^. Fgfr2^C342Y/+^ and wild type mice on the Balb/C genetic background were utilized for cell isolations and for micro-CT analyses. NIH III nude mice were obtained from Charles River Laboratories International, Inc. (Wilmington, MA) and utilized as donor mice for subcutaneous implant experiments. All animal procedures were performed according to University of Michigan's University Committee on Use and Care of Animals.

### 2.2. Microcomputed Tomography

Tibias of Fgfr2^C342Y/+^ (*n* = 14) and wild type (*n* = 14) four-week-old mice were embedded in 1% agarose and scanned at the proximal metaphysis and the mid-diaphysis using a microcomputed tomography imaging system (Scanco *µ*CT100, Scanco Medical, Bassersdorf, Switzerland). Scan settings were voxel size 12 *µ*m, 70 kVp, 114 *µ*A, 0.5 mm AL filter, and integration time of 500 ms. Density measurements were calibrated to the manufacturer's hydroxyapatite phantom. Analysis was performed using the manufacturer's evaluation software (Scanco *µ*CT100, Scanco Medical, Bassersdorf, Switzerland) using a fixed global threshold of 28% (280 on a grayscale of 0–1000) to segment bone from nonbone. Micro-CT bone data was analyzed and is reported in accordance with the recommendations of Bouxsein et al., 2010 [34]. Statistical significance between groups was established by use of the Student's *t*-test.

### 2.3. Primary Cell Isolation

Bone marrow stromal cells were isolated from the long bones of four-week-old Crouzon mice and wild type littermates, as previously described [35]. Briefly, marrow cells were aspirated using a 25-gauge needle and a 5 mL syringe containing media. Marrow was flushed and cells were then dispersed by aspirating several times through a 22-gauge needle. Cells were cultured in *α*MEM supplemented with 20% fetal bovine serum (FBS) and 10,000 *μ*g/mL penicillin/streptomycin for several days. Media was changed every three days until all suspension cells were removed and adherent cells were confluent. 

### 2.4. *In Vitro* Osteoblast Differentiation

Cells were induced to differentiate *in vitro* by culture in media containing 50 *μ*g/mL ascorbate for the indicated number of days. RNA was isolated using Trizol reagent (Invitrogen) following manufacturer protocols. mRNA levels were assayed by reverse transcription and real-time PCR. Real-time PCR was performed utilizing the murine tissue nonspecific alkaline phosphatase (TNAP) primer/probe set Mm00475834_m1, the murine bone sialoprotein (BSP) primer/probe set Mm00492555_m1, the murine osteocalcin (OCN) primer/probe set Mm03413826_mH, the murine runt related transcription factor 2 (Runx2) primer/probe set Mm00501578_m1, the murine collagen type I, alpha 1 (col1a1) primer/probe set Mm00801666_g1, the murine hypoxanthine phosphoribosyltransferase-1 (Hprt1) primer/probe set Mm01545399_m1, and Taqman Universal PCR Master Mix (Applied Biosystems). Real-time PCR was performed on a GeneAmp 7700 thermocycler (Applied Biosystems) and quantified by comparison to a standard curve. mRNA levels are reported after normalization to Hprt1 mRNA levels. Cells were induced to form mineral by addition of 10 mM *β*-glycerophosphate. Mineralized nodules were stained by Von Kossa. Briefly, cells were rinsed with phosphate-buffered saline, fixed with 100% ethanol and rehydrated in a graded ethanol series. Cells were then incubated in 5% AgNO_3_, rinsed with dH_2_O and exposed to light for 1 hour. For quantification, wells were scanned and densitometry was measured using *NIH Image* software. Tissue non-specific alkaline phosphatase (AP) enzyme activity was assayed using the colorimetric substrate, NBT/BCIP (Sigma). Cells were fixed in 70% ethanol for 10 minutes at room temperature, air dried, and incubated with substrate for 1 hour at 37C. Cells were then rinsed with dH_2_O, air dried, and visualized macroscopically for evidence of staining. For quantification, wells were scanned and densitometry was measured using *NIH Image* software. Statistical significance between genotypes for mRNA levels, quantification of mineralization, and quantification of AP enzyme activity was established by use of the Student's *t*-test. 

### 2.5. Subcutaneous Implant Preparation and Analysis of Ossicles

**Figure 1:**
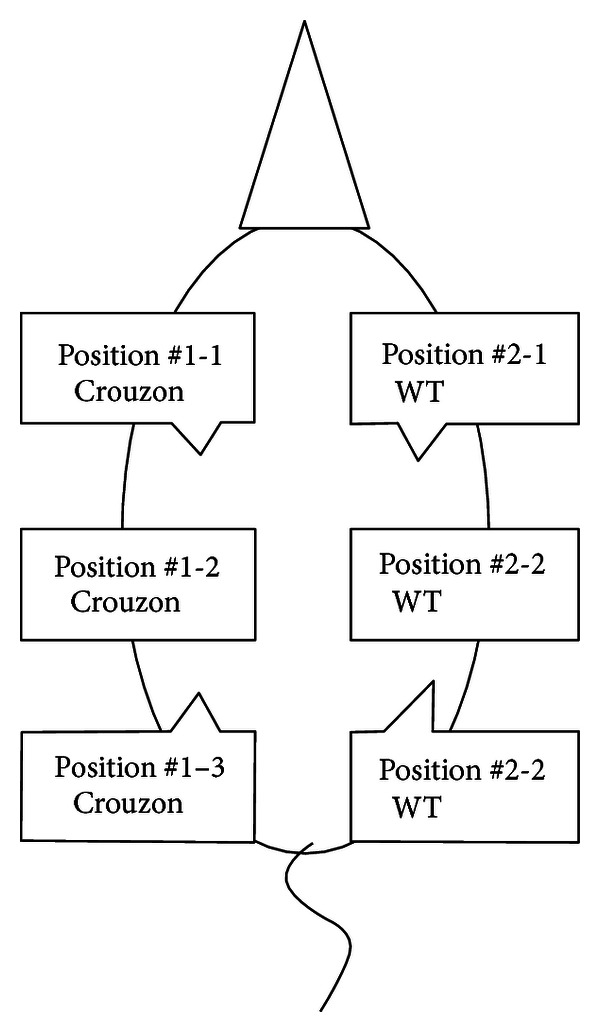
Subcutaneous implant placement. This schematic shows the position of six implants that were placed subcutaneously, on the dorsal surface of immunodeficient mice.

Subcutaneous implants were prepared as previously described [36]. 4 × 10^7^ bone marrow stromal cells were mixed with 0.01% NaOH in phosphate buffered saline and 1ml of rat tail collagen solution (BD Biosciences, San Jose, CA) on ice. The solution was aliquoted into glass tissue culture wells (*Lab-Tek* 16 well chamber slide system; Nalge Nunc International, Rochester, NY) and then incubated at 37°C for 1 hour to allow for gel hardening. Midline longitudinal incisions were made along the dorsal surface of each six-week-old NIH III mouse, and subcutaneous pockets were formed laterally, by gentle blunt dissection. A single implant was placed into each subcutaneous pocket, for a total of six implants per animal (Figure 1). Implants were removed eight weeks after implantation and analyzed for mineralized tissue formation by radiography (Faxitron MX-20, Faxitron Bioptics LLC, Tucson, AZ). All implants were imaged on the same film. Mineralized tissue of twelve implants from each genotype was quantified by densitometry (ImageJ, NIH). Statistical significance between genotypes was established by use of the Student's *t*-test. Implants were then homogenized for alkaline phosphatase measurements or decalcified and embedded in paraffin for histologic analysis by trichrome or hematoxylin and eosin staining. Alkaline phosphatase enzyme activity of implants was measured by homogenizing the implants in a solution containing 1.6 M MgCl_2_, 0.2 M Tris-Cl pH 8.1, and 1% triton X-100 followed by incubation of lysate with 7.5 mM of 4-nitrophenyl-phosphate at room temperature for 1 hour. Absorbance at 405 nm was measured and results were normalized to DNA content. Statistical significance between genotypes was established by use of the Student's *t*-test.

## 3. Results

### 3.1. Animals

The Crouzon Fgfr2^C342Y/+^ mutant mice show a phenotype similar to that which was previously reported [5, 32, 37]. The mutant mice are slightly smaller in body size than their wild type littermates and exhibit craniofacial abnormalities associated with craniosynostosis including a dome-shaped skull, wide set and proptotic eyes, and severe midface hypoplasia. 

### 3.2. *In Vitro* Osteoblast Differentiation

**Figure 2:**
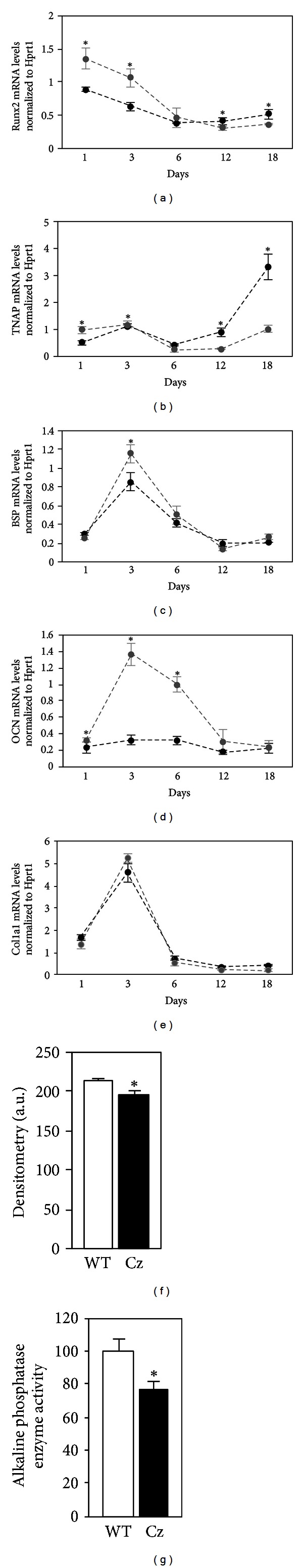
The Fgfr2^C342Y^ mutant bone marrow stromal cells exhibit abnormal osteoblastic gene expression and diminished mineralization *in vitro*. Bone marrow stromal cells were isolated from Crouzon Fgfr2^C342Y/+^ (Cz) and wild type (WT) littermates and then cultured with ascorbate for the indicated number of days to induce osteoblast differentiation. Runx2, bone sialoprotein (BSP), osteocalcin (OCN), and tissue non-specific alkaline phosphatase (TNAP) and collagen type 1 alpha1 (col1a1) mRNA levels were measured by real-time PCR. Black lines represent wild type; grey lines represent Crouzon (a, b, c, d, and e). Results are presented as normalized to Hprt1. Cells were cultured with ascorbate and *β*-glycerophosphate to induce mineralization for 18 days (f). Mineralized nodules were stained by Von Kossa and quantified by densitometry. Cells were cultured with ascorbate for 18 days, and alkaline phosphatase (Tnap/Alpl/Akp2) enzyme activity was quantified by incubation of cells with a colorimetric substrate. Enzyme activity was quantified by densitometry (g). Results shown are means ± standard deviations from triplicate experiments for all data shown. *P < .05 between genotypes.

Analysis of mRNA levels demonstrates that bone marrow stromal cells isolated from Crouzon mice express significantly higher levels of Runx2 and tissue non-specific alkaline phosphatase enzyme (AP) at days 1 and 3 of differentiation, significantly higher levels of bone sialoprotein at day 3 of differentiation, and significantly higher levels of osteocalcin at days 1, 3, and 6 of differentiation (Figures 2(a), 2(b), 2(c), and 2(d)). This data indicates that the Fgfr2^C342Y^ mutation enhances the expression of osteoblastic genes in bone marrow stromal cells during early stages of osteoblast differentiation. In contrast, Runx2 and tissue non-specific alkaline phosphatase mRNA levels were significantly lower in cells isolated from Crouzon mice at days 12 and 18 of differentiation (Figures 2(a) and 2(b)). Cells isolated from Crouzon mice also exhibited less alkaline phosphatase enzyme activity at day 18 of differentiation and mineralized to a diminished extent than cells isolated from wild type littermates (Figures 2(f) and 2(g)). In combination, this data suggests that the Fgfr2^C342Y^ mutation may inhibit later stages of osteoblast differentiation and inhibit mineralization. Notably, collagen type 1, alpha 1 mRNA expression levels were not different between Crouzon and wild type cells at any stages of differentiation. This data suggests that the diminished mineralization by Crouzon cells is not likely due to diminished collagen expression. Together, these results indicate that while the Fgfr2^C342Y^ mutation may enhance early osteoblast differentiation, it also appears to inhibit expression of tissue non-specific alkaline phosphatase and mineralization by more differentiated cells. The findings also indicate that the Fgfr2^C342Y^ mutation induces autonomous abnormalities in osteoblast differentiation when bone marrow stromal cells are cultured in a conventional two-dimensional monolayer system *in vitro*. 

### 3.3. *In Vivo* Mineralized Tissue Formation

**Figure 3:**
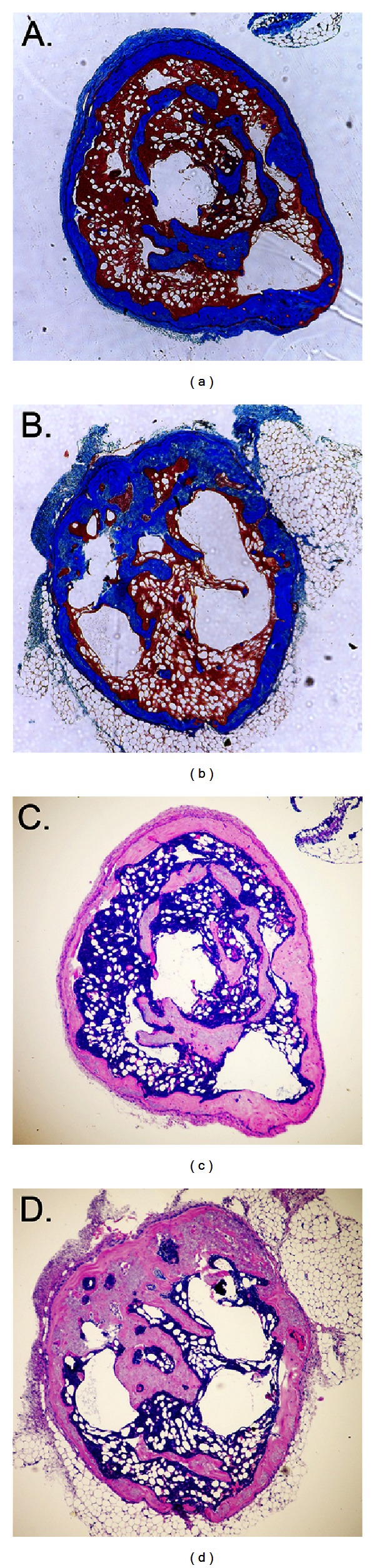
Histologic staining of implants. Ossicles formed eight weeks after subcutaneous implantation of cells mixed with a collagenous matrix were stained by trichrome (a and b) or by hematoxylin and eosin (c and d). Note the greater amount of deep blue (a) and light pink (c) staining in ossicles formed by wild type cells, indicative of greater bone formation by these cells. In comparison, note the greater amount of light blue (b) and greyish-pink (d) staining in ossicles formed by Crouzon cells, indicative of greater amounts of original implanted collagen. Also note that ossicles formed by either wild type or mutant cells contain bone marrow.

**Figure 4:**
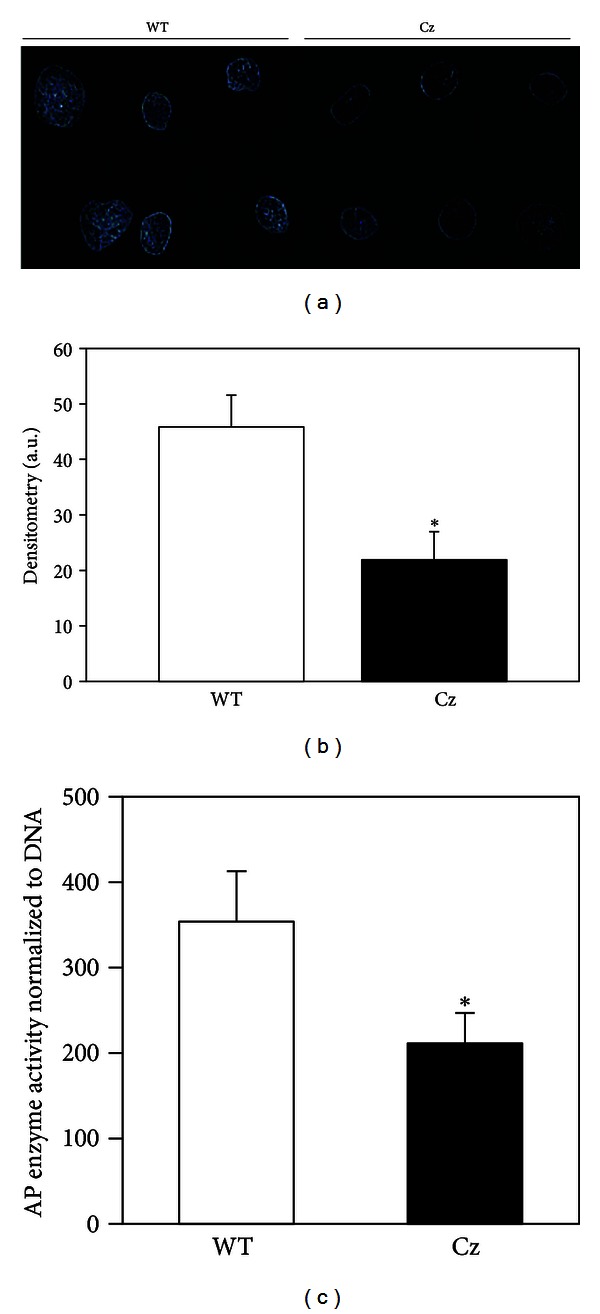
The Fgfr2^C342Y^ mutation inhibits mineralized tissue formation *in vivo*. (a) Radiographic image of representative implants shows increased radiodensity of ossicles formed by wild type (WT), as compared to those formed by Crouzon (Cz) cells. (b) Quantification of radiodense tissue by densitometry confirms that implanted wild type cells formed significantly greater amounts of mineralized tissue compared to mutant cells (*n* = 12 implants per genotype). *P < .05 versus WT. (c) Homogenized implants formed by wild type cells also have significantly greater levels of alkaline phosphatase enzyme expression than homogenized implants formed by Crouzon cells (*n* = 3 implants per genotype). *P < .05 versus WT.

Because a three-dimensional matrix promotes a more physiologically relevant cellular state than conventional *in vitro* monolayer cell culture [33, 38–40], we next assayed the cells when allowed to differentiate in a collagenous matrix *in vivo*. In accordance with the *in vitro* data, bone marrow stromal cells isolated from Crouzon mice formed significantly less mineralized tissue than cells isolated from wild type mice, when allowed to differentiation in a three-dimensional collagenous matrix *in vivo*. Both trichrome (bone tissue stains deep blue and implanted collagen stains lighter blue) and H&E histologic stains (bone tissue stains pink and implanted collagen stains greyish-pink) indicate that the ossicles formed by Crouzon cells may contain less mineralized tissue than ossicles formed by wild type cells (Figure 3). Notably, the marrow of implants prepared using either Crouzon or wild type cells contains both hematopoietic and adipocytic cells (small empty ovals are adipocyte ghosts). This may indicate that the Fgfr2^C342Y^ mutation does not influence hematopoietic or adipocytic cell differentiation, although more comprehensive analyses of these cells types in both heterozygous and homozygous mutant mice are required to definitely determine if this is the case. Radiographs of representative implants dissected eight weeks after implantation reveal apparently diminished radiodensity of ossicles prepared using cells isolated from the mutant, as compared to the wild type mice (Figure 4(a)). Quantification of mineralized tissue confirms that the mutant cell implants have significantly less mineralized tissue than the wild type cell implants (Figure 4(b)). Finally, alkaline phosphatase enzyme expression, which is essential for mineral deposition in collagenous tissues [41] was found to be significantly lower in implants prepared using mutant than wild type cells (Figure 4(c)). Together, these results demonstrate that the Fgfr2^C342Y^ mutation inhibits alkaline phosphatase enzyme expression and the formation of mineralized tissue by osteoprogenitor cells when allowed to differentiate in a three-dimensional matrix *in vivo*.

### 3.4. Microcomputed Tomographic Analysis of Long Bones

**Figure 5:**
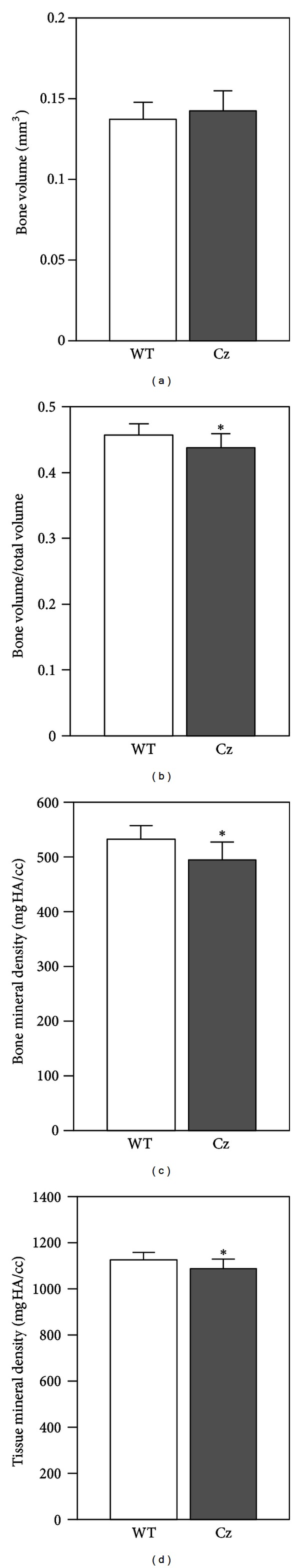
Diminished cortical bone volume and density in the long bones of Fgfr2^C342Y/+^ mice. Micro-CT analyses demonstrate significantly diminished cortical bone volume/total volume, bone mineral density, and tissue mineral density in tibias of Fgfr2^C342Y/+^ (Cz) mice as compared to wild type (WT) mice. *P < .05 between genotypes.

**Figure 6:**
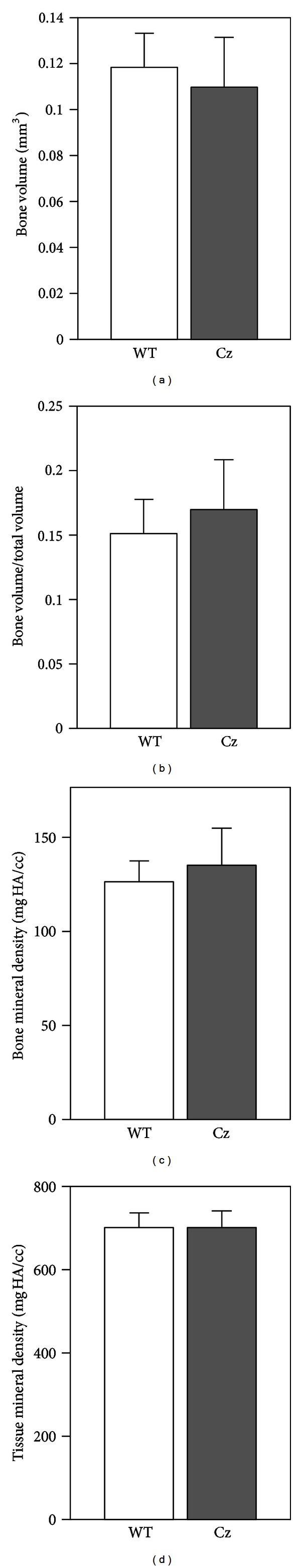
Similar trabecular bone volume and density in the long bones of Fgfr2^C342Y/+^ mice. Micro-CT analyses demonstrate no significant differences in bone volume, bone volume/total volume, bone mineral density, and tissue mineral density in tibias of Fgfr2^C342Y/+^ (Cz) mice as compared to wild type (WT) mice.

We recently showed that the frontal cranial bones of Fgfr2^C342Y/+^ mice are diminished in bone volume and density when compared to those of wild type mice [5]. To determine if bones of the appendicular skeleton are similarly affected, we utilized microcomputed tomography to analyze parameters of bone quality and quantity in tibias of four-week-old Fgfr2^C342Y/+^ and Fgfr2^+/+^ mice. Results demonstrate that Fgfr2^C342Y/+^ mice have significantly diminished tibial cortical bone volume/total volume, bone mineral density, and tissue mineral density (Figure 5), when compared to FGFR2^+/+^ mice. Trabecular measures of tibial bone volume/total volume, bone mineral density, and tissue mineral density were not different between Fgfr2^C342Y/+^ and Fgfr2^+/+^ mice (Figure 6). These results demonstrate that the Fgfr2^C342Y/+^ mutation is associated with decreased bone volume and density that is not limited to the craniofacial skeleton.

## 4. Discussion

The pathogenesis of craniosynostosis remains unknown and until this knowledge has been realized, the only treatment for craniosynostosis will remain that of surgical intervention. While it is not known if craniosynostosis results primarily from abnormalities in cranial bone cells, cranial suture cells and/or abnormalities in other cell types, mounting evidence does indicate that craniosynostosis occurs in the context of diminished cranial bone quantity and quality. Fgfr2^S250W^ Apert mice and  Fgfr3^P244R^ Muenke mice were both previously shown to exhibit craniosynostosis in combination with lower levels of bone formation and/or mineralization when compared to wild type littermates [42, 43]. As noted previously, Fgfr2^C342Y/C342Y^ Crouzon mice were originally characterized as having decreased ossification of vertebral bodies [32]. In addition, we recently reported that Fgfr2^C342Y/+^ mice on a BALB/c genetic background have diminished cranial bone volume and density when compared to wild type mice [5]. Abnormal BMP signaling in neural crest cells was also recently shown to cause diminished cranial bone volume and density in combination with craniosynostosis [44]. Taken together, these results appear to indicate that craniosynostosis is an abnormality involving excessive ectopic mineralization (bone formation at a temporally and spatially inappropriate location, such as the juvenile cranial suture) and not a disorder of excessive eutopic bone formation. In fact, craniosynostosis associated with several distinct genetic mutations (cited earlier) appears to occur in combination with diminished eutopic bone mass. This distinction is critical for the future development of biologically based therapeutics for the prevention and/or treatment of craniosynostosis.

Here we report that bone marrow stromal cells isolated from Crouzon Fgfr2^C342Y/+^ mice express significantly higher levels of some osteoblastic genes during early stages of differentiation but significantly lower levels of tissue non-specific alkaline phosphatase mRNA and enzyme activity, as well as diminished mineralization when cells are further differentiated in a 2D *in vitro* cell culture system. These results are in accordance with our previous report which showed that frontal bone cells isolated from Fgfr2^C342Y/+^ mice exhibited enhanced early osteoblastic differentiation but diminished later stage differentiation and a decreased tendency to form mineralized tissue, when compared to cells isolated from wild type mice *in vitro* [5]. That the Fgfr2^C342Y^ mutation stimulates early osteoblast differentiation while inhibiting later maturation into fully functional osteoblasts could explain the apparently discrepant results found in the literature regarding effects of craniosynostosis-associated FGFR mutations on osteoblast differentiation. Our *in vitro* data, showing that Crouzon Fgfr2^C342Y^ marrow stromal cells express higher levels of multiple osteoblastic genes than wild type cells during early stages of differentiation, is in accordance with previous reports which showed increased osteoblastic gene expression in cranial suture tissues. Previous *in vivo* analyses of the Fgfr2^P253R/+^ and Fgfr2^S252W/+^ mouse models of Apert syndrome revealed increased osteoblastic gene expression around the coronal suture [45, 46]. Mice carrying the P250R mutation in Fgfr1 associated with Pfeiffer syndrome were also previously shown to exhibit enhanced osteoblastic differentiation of cells within the sagittal suture [47]. Finally, newborn Fgfr2^C342Y/+^ mice were also previously shown to have increased Runx2 mRNA levels around the coronal suture, as compared to wild type littermates [32]. Increased expression of osteoblastic genes in suture tissues is well reconciled with our results showing increased osteoblastic gene expression in Fgfr2^C342Y/+^ bone marrow stromal cells during early stages of differentiation. In contrast, our data showing that Fgfr2^C342Y^ marrow stromal cells express significantly lower levels of Runx2 and tissue non-specific alkaline phosphatase mRNA, as well as significantly diminished alkaline phosphatase enzyme activity and mineralization, is in accordance with previous studies showing that S252W, C342Y, and P253R craniosynostosis-associated mutations in FGFR2 inhibit osteoblast differentiation [48–50]. The data can also potentially explain the diminished eutopic bone mass that is seen in multiple mouse models of craniosynostosis, including the Fgfr2^C342Y^ mouse model of Crouzon syndrome. Taken together, it appears that craniosynostosis syndrome-associated mutations in Fgfr's enhance or inhibit osteoblast differentiation in a cell type, environment, and differentiation stage dependent manner.

Importantly, two-dimensional cell culture on plastic does not well represent the environmental conditions that cells find in physiologic tissues, and it has been suggested that three-dimensional cell culture helps to “bridge the gap” between cultured cells and the *in vivo* environment [33]. Therefore, to increase confidence in our *in vitro* findings, we also mixed bone marrow stromal cells in a three dimensional collagenous matrix and allowed them to differentiate when implanted into donor mice. Results of these experiments showed that the Crouzon Fgfr2^C342Y^ mutation inhibited bone formation and alkaline phosphatase enzyme expression, again supporting the idea that the Fgfr2^C342Y^ mutation inhibits later stage osteoblast differentiation and bone formation. While our results also show significantly diminished bone volume and density in the long bones of Fgfr2^C342Y^ mice, future studies are required to definitively establish that Crouzon bone marrow stromal cells are deficient in their ability to differentiate into fully functional osteoblasts and form bone *in vivo*. If correct, our results suggest that patients carrying craniosynostosis syndrome associated Fgfr mutations may be at higher risk for osteoporosis and/or slow repair of long bone fractures. 

While the diminished cranial bone formation in these Fgfr-associated mouse models of craniosynostosis has not been previously considered as contributing to the development of craniosynostosis, it is interesting to note that craniosynostosis is also known to occur in other disorders of low bone mineralization. Mutations in the phosphate regulating protein Phex cause X-linked hypophosphatemic rickets involving low serum phosphate, defective bone mineralization and also craniosynostosis [51–53]. It is unknown how Phex mutations lead to craniosynostosis, but, similar to studies of human patients with Fgfr2-associated craniosynostosis [30], these patients also commonly have paradoxical heterotopic calcification of normally nonmineralizing tissues, such as tendons and ligaments [53]. Craniosynostosis is also seen in infantile hypophosphatasia due to inactivating mutations in the enzyme, tissue non-specific alkaline phosphatase (TNAP) [54–56]. These patients have severely deficient bone mineralization [57]. TNAP is an enzymatic generator of inorganic phosphate and an established essential promoter of tissue mineralization, but it is again unknown how diminished TNAP activity leads to craniosynostosis [41, 58]. Finally, craniosynostosis was also previously reported to occur secondary to antacid-induced infantile hypophosphatemia [59]. This data indicates that craniosynostosis occurs in multiple disorders involving dysregulated phosphate homeostasis and bone mineralization. Notably, signaling through Fgfr's was also previously shown to regulate expression of enzymes that control the local production of inorganic phosphate [35], and here we show that cells isolated from Crouzon mice express significantly lower levels of tissue non-specific alkaline phosphatase mRNA and significantly diminished alkaline phosphatase enzyme expression. Together, these results make it tempting to hypothesize that craniosynostosis may be promoted by abnormal tissue levels of phosphate leading to aberrant tissue mineralization.

In conclusion, this study demonstrates that the Crouzon syndrome associated C342Y mutation in Fgfr2 enhances early osteoblast differentiation but inhibits later differentiation of bone marrow stromal cells into fully functional osteoblasts when cultured in a conventional *in vitro* monolayer system and when allowed to differentiate in a three-dimensional matrix *in vivo*. This study also demonstrates that the long bones of Fgfr2^C342Y^ mice have significantly diminished bone volume and density when compared to wild type littermates. Taken together, our results indicate that Crouzon cells have an intrinsic or cell autonomous defect in osteoblast differentiation and bone formation that includes cells of the appendicular skeleton. Future studies are required to determine if Crouzon syndrome patients are at increased risk for osteoporosis and/or poor repair of bony fractures due to this abnormal cell behavior.
